# Comprehensive analysis on clinical significance and therapeutic targets of LDL receptor related protein 11 (LRP11) in liver hepatocellular carcinoma

**DOI:** 10.3389/fphar.2024.1338929

**Published:** 2024-02-15

**Authors:** Wonbeak Yoo, Ae-Kyeong Kim, Hae Un Kook, Kyunghee Noh

**Affiliations:** ^1^ Personalized Genomic Medicine Research Center, Korea Research Institute of Bioscience and Biotechnology (KRIBB), Daejeon, Republic of Korea; ^2^ Bionanotechnology Research Center, Korea Research Institute of Bioscience and Biotechnology (KRIBB), Daejeon, Republic of Korea; ^3^ Metabolic Regulation Research Center, Korea Research Institute of Bioscience and Biotechnology (KRIBB), Daejeon, Republic of Korea; ^4^ Department of Functional Genomics, KRIBB School of Bioscience, Korea University of Science and Technology (UST), Daejeon, Republic of Korea; ^5^ Department of Nanobiotechnology, University of Science and Technology (UST), Daejeon, Republic of Korea

**Keywords:** Lrp11, liver hepatocellular carcinoma, prognosis, immune infiltrates, epigenetic regulation

## Abstract

LDL lipoprotein receptor-related protein 11 (LRP11) plays a role in several tumors. However, their roles in hepatocellular carcinoma remain unclear. The present study aimed to explore the expression profile and prognostic value of LRP11 in liver hepatocellular carcinoma (LIHC) patients using various cancer databases and bioinformatic tools. In bioinformatics analysis, The Cancer Genome Atlas datasets showed increased LRP11 expression in tumor tissues compared to that in non-tumor tissues in various cancers. Moreover, patients with high expression LRP11 correlated with poor prognosis and clinical features. The LRP11 expression positively correlated with the infiltration of immune cells such as macrophages, neutrophils, and myeloid-derived suppressor cells and a combination of high LRP11 expression and high immune infiltrates was associated with the worst survival in LIHC tumors. Our results also indicated that LRP11 expression was closely associated with immune-modulate function, such as antigen presentation. In DNA methylation profiling, hypomethylation of LRP11 is widely observed in tumors and has prognostic value in LIHC patients. Functional enrichment analysis revealed that LIHC-specific LRP11 interacting genes are involved in protein binding, intracellular processing, and G-protein-related signaling pathways. Analyses of drug sensitivity and immune checkpoint inhibitor predict a number of drugs that could potentially be used to target LRP11. In addition, *in vitro* experiments verified the promoting effect of LRP11 on the migration, invasion, and colony formation capacity of hepatocellular carcinoma cells. Collectively, our results aided a better understanding of the clinical significance of LRP11 in gene expression, functional interactions, and epigenetic regulation in LIHC and suggested that it may be a useful prognostic biomarker for LIHC patients.

## Introduction

In recent years, cancer has become one of the main causes of mortality worldwide, and its incidence has been gradually increasing, with a significant negative impact on human health and social development (Siegel et al., 2022). Liver hepatocellular carcinoma (LIHC) is the most common primary liver cancer and the third leading cause of cancer-related deaths worldwide (Rumgay et al., 2022). Despite extensive cancer diagnosis, treatment, and molecular characterization research in LIHC patients, overall recurrence and mortality rates remain high (Heimbach et al., 2018; Sung et al., 2021). Poor prognosis and clinical progression are related to the fact that most LIHC cases may not be diagnosed in the early stages (Cervello et al., 2020). Hence, there is an urgent need to identify reliable biomarkers to explain the molecular mechanisms of LIHC incidence and improve prognosis.

Low-density lipoprotein receptor-related protein (LRP11) is a member of low-density lipoprotein receptor (LDLR) family member (Roslan et al., 2019). The LDLR family consists of transmembrane proteins that encode single-span transmembrane receptors, usually called LDLR-related proteins (LRP) (Campion et al., 2020). LDLR is associated with various cancers, including liver, leukemia, lung, breast, colorectal, and prostate (Huang et al., 2016; Kimbung et al., 2016). Among the LDLR family members, LRP11 was recently identified as a prognostic marker and therapeutic target in prostate and cervical cancers (Wang et al., 2019; Gan et al., 2020; Gu et al., 2023); however, the underlying association and role of LRP11 in LIHC remain unknown.

This is the first study to comprehensively investigate the association between LRP11 and LIHC. In this study, we investigated the expression of LRP11 in LIHC and its relationship with prognosis and clinicopathological parameters. We also examined the effect of LRP11 expression on tumor microenvironment and epigenetic profiling and explored the role of LRP11 in gene networks and biological functions. Further *in vitro* experiments evidenced that LRP11 regulated the potential of cell migration, invasion, and colony formation of LIHC cells, and it also involved in the regulation of epithelial-mesenchymal transition (EMT).

Our results demonstrated the importance of LRP11 in determining the prognosis of LIHC. They showed that LRP11 expression may be regulated by epigenetic differences related to prognosis and is associated with cancer immunity. Collectively, our study suggested that LRP11 is a significant prognostic biomarker and a new treatment target for LIHC.

## Materials and methods

### Bioinformatic analysis of LRP11 expression from public database

The GEPIA database was used to compare LRP11 expression between various cancers including LIHC and normal tissues, as well as access overall survival (OS) and disease-free survival (DFS) based on the expression of LRP11. This database was also used to further associations between LRP11 and the expression of immune-related marker genes have been verified. The Gene Expression Omnibus (GEO, https://www.ncbi.nlm.nih.gov/geo/) dataset (GSE25097, GSE36376, GSE36411, GSE45436, GSE54236, and GSE76427) was used as LIHC validation sets. The KM plotter database was applied to analyze the prognostic value of LRP11 in LIHC (Lanczky and Gyorffy, 2021). The correlation between LRP11 and clinicopathological feature was explored using UALCAN database. OSdream database was used to predict whether LRP11 has a risk of recurrence of LIHC patients, and clinical feature with univariate and multivariate Cox regression prognostic values were included in the nomogram analysis. BEST tool (https://rookieutopia.com/) was used to predict the clinical association including radiotherapy and sorafenib treatments, immunomodulation related gene, and candidate agents in patients with LIHC. To evaluate the therapeutic potential of LRP11 in a variety of cancer cell lines, shinyDepMap was used to analyze Cancer Dependency Map (DepMap) datasets (Shimada et al., 2021).

### Analysis of microenvironmental characteristics in LIHC

The expression of LRP11 in malignant and non-malignant cells in LIHC was analyzed Human Liver Browser and Single-cell Atlas in Liver Cancer (scAtlasLC) (Ma et al., 2021). The Tumor Immune Estimation Resource Database (TIMER 2.0) used to characterize immune-infiltrates and visualization of TCGA in the TIMER database (Li et al., 2020). This database also used to analyze the LIHC-infiltrating immune cells with LRP11 gene expression and strength of correlations.

### LRP11 methylation analysis

The CpG methylation (β-values) associated with LRP11 in TCGA-LIHC and Heatmap analysis were evaluated using platform MethSurv (Modhukur et al., 2018). Moreover, Shiny Methylation Analysis Resource Tool (SMART) was used to analyze differential methylation by each LRP11 probe and Spearman’s correlation between methylation level and LRP11 expression. The significant CpGs were classified according to their functional roles in genomic locations such as promoters within 1,500 bps of a transcription start site (TSS) (TSS1500); within 200 bps of a TSS (TSS200); 5′ untranslated regions (5′UTR); first exon (1stExon); body (non-promoter); 3′UTR (non-promoter). The OS analysis for each CpG site was assessed using KM plots. Log-rank tests were used to measure the statistical significance and Log-rank *p* < 0.05 was considered significant.

### Gene enrichment analysis and network construction

Using Pathway Commons database, we selected 16 genes with the strongest correlation with LRP11, which allowed to generate a protein-protein interaction (PPI) network for the LRP11 gene as well as binding and target genes. The common genes were then used for further analysis using PANTHER database (http://pantherdb.org/) for constructing pathways in molecular function, biological process, and pathway in this study.

### Cell culture

Cell culture and RNA extraction were performed as described (Yoo et al., 2023). Briefly, Huh7 and Hep3B, and HepG2, and FOCUS were maintained in Dulbecco’s modified Eagle’s medium containing 10% FBS (GIBCO, Grand Island, NY, USA). HepaRG cells were grown in William’s E medium supplemented with 10% of FBS, 5 μg/mL insulin, 2-mM Glutamax, 1% penicillin–streptomycin, and 50 µM hydrocortisone hemisuccinate (Sigma-Aldrich) in a humidified incubator with 5% CO_2_ at 37°C.

### Real-time quantitative reverse transcription-polymerase chain reaction (qRT-PCR) analysis

Total RNA was extracted using a TRIzol reagent-based kit (Intron Biotech, Seongnam-Si, Republic of Korea). cDNA was reverse transcribed with the SuperScript IV First-Strand Synthesis System for RT-PCR (Thermo Fisher Scientific, Waltham, MA, United States) according to the manufacturer’s protocol. cDNA was amplified using the reported primers (Supplementary Table S1) and SYBR Premix Ex Taq (Takara Bio, Otsu, Shiga, Japan and Agilent Technologies, Santa Clara, CA, United States). The relative mRNA levels were detected by qPCR with the manufacturer’s instructions (Applied Biosystems, Foster City, CA, United States, and Agilent Technologies). The relative quantification of gene expression was performed using the 2^−ΔΔCT^ method.

### Cell migration assay and matrigel invasion assay

After transfection of 48 h with hLRP11 siRNA (Bioneer, Republic of Korea, CAT # SDO-1001), HepG2 and Hep3B cells in RPMI with 0.5% serum were seeded into the upper chamber of the transwell. The insert was then placed in a 24-well plate containing RPMI with 10% serum in the lower chamber as a chemoattractant. After cells were allowed to migrate for 24 h in a humidified chamber, those that had migrated were stained with a 0.5% crystal violet (w:v) 20% methanol and counted by light microscopy in five random fields (×100 original magnification) per sample. For the Matrigel invasion assay, the insert was coated with a thin layer of 0.5 mg/mL Matrigel Basement Membrane Matrix (BD Biosciences). siRNA LRP11 transfected HepG2 and Hep3B cells were placed in the upper chamber with 0.5% serum containing RPMI medium, and 0.5 mL of growth medium containing 10% FBS was placed in the lower chamber. The cells were incubated at 37°C and allowed to invade through the Matrigel layer for 48 h. The invading cells on the lower surface were stained with 0.5% crystal violet (w:v) 20% methanol and stained cells were counted under the light microscopy.

### Cell colony formation assay

siRNA Control (siControl) or siRNA LRP11 (siLRP11) transfected HepG2 and Hep3B cells (500 cells/well) were seeded in a 6-well plate and cell culture medium was replaced ever 2–3 days for 10 days. The colonies appeared in the 6-well plates, cells were washed twice with PBS. Next, the cells were fixed with methanol for 15 min and stained with 0.5% crystal violet (w:v) 20% methanol for 10 min. The number of cell colonies were counted.

### Statistical analysis

The statistical analysis was calculated automatically based on the online database above. Student’s t-test implemented by GraphPad Prism (Version 9). Correlations were analyzed by Spearman and Pearson’s correlation. *p* < 0.05 was considered statistically significant.

## Results

### Expression and prognostic value of LRP11 in various human cancer

**FIGURE 1:**
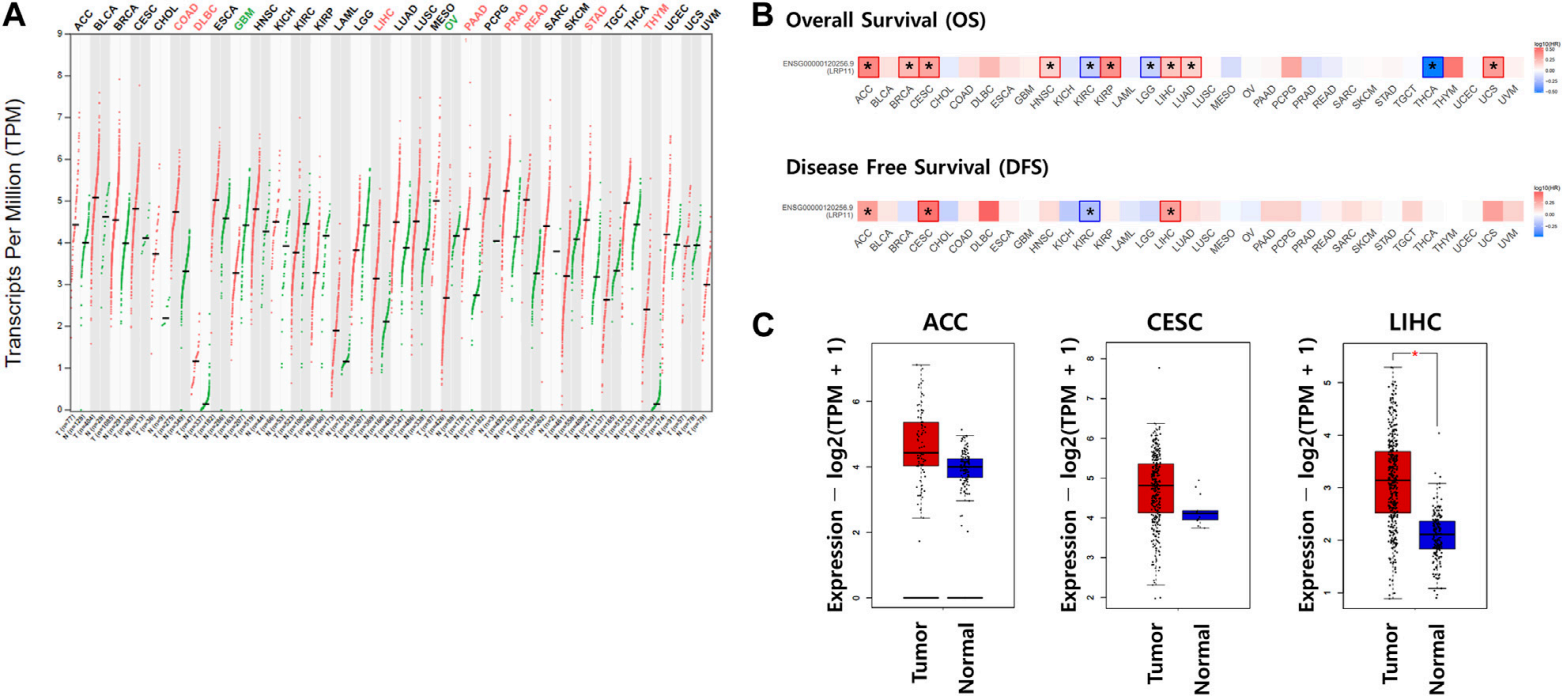
The impact of LRP11 expression on prognosis in various human cancers. **(A)** Expression levels of LRP11 in GEPIA database. **(B)** Prognostic value of LRP11 in various cancers. **(C)** Transcription levels of the LRP11 gene using GEPIA based on the TCGA and GTEx database. Normal tissues are matched TCGA adjacent tissue and GTEx data. ACC (tumor n = 77, normal n = 128), CESC (tumor n = 306, normal n = 13), and LIHC (tumor n = 369, normal n = 160) samples, respectively. The method for differential analysis is one-way ANOVA. ACC, Adrenocortical carcinoma; BLCA, Bladder Urothelial Carcinoma; BRCA, Breast invasive carcinoma; CESC, Cervical squamous cell carcinoma and endocervical adenocarcinoma; CHOL, Cholangiocarcinoma; COAD, Colon adenocarcinoma; DLBC, Lymphoid Neoplasm Diffuse Large B cell Lymphoma; ESCA, Esophageal carcinoma; GBM, Glioblastoma multiforme; HNSC, Head and Neck squamous cell carcinoma; KICH, Kidney Chromophobe; KIRC, Kidney renal clear cell carcinoma; KIRP, Kidney renal papillary cell carcinoma; LAML, Acute Myeloid Leukemia; LGG, Brain Lower Grade Glioma; LIHC, Liver hepatocellular carcinoma; LUAD, Lung adenocarcinoma; LUSC, Lung squamous cell carcinoma; MESO, Mesothelioma; OV, Ovarian serous cystadenocarcinoma; PAAD, Pancreatic adenocarcinoma; PCPG, Pheochromocytoma and Paraganglioma; PRAD, Prostate adenocarcinoma; READ, Rectum adenocarcinoma; SARC, Sarcoma; SKCM, Skin Cutaneous Melanoma; STAD, Stomach adenocarcinoma; TGCT, Testicular Germ Cell Tumors; THCA, Thyroid carcinoma; THYM, Thymoma; UCEC, Uterine Corpus Endometrial Carcinoma; UCS, Uterine Carcinosarcoma; UVM, Uveal Melanoma.

Using the GEPIA2 database, mRNA expression of LRP11 was investigated in all cancers. As shown in Figure 1A, higher expression of LRP11 was observed in colon adenocarcinoma, lymphoid neoplasm diffuse large B cell lymphoma, liver hepatocellular carcinoma, pancreatic adenocarcinoma, prostate adenocarcinoma, rectum adenocarcinoma, stomach adenocarcinoma, and thyroid carcinoma. In contrast, LRP11 expression decreases in glioblastoma multiforme and ovarian serous cystadenocarcinoma. To explore the prognostic significance of LRP11, overall survival (OS) and disease-free survival (DFS) were investigated in a pan-cancer analysis. Patients with higher LRP11 levels had worse OS than that of patients with lower LRP11 levels in adrenocortical carcinoma (ACC, HR:2.5; 95% CI; Logrank-p = 0.02), breast invasive carcinoma (BRCA, HR:1.7; 95% CI; Logrank-p<0.001), cervical squamous cell carcinoma and endocervical adenocarcinoma (CESC, HR:2; 95% CI; Logrank-p = 0.0049), head and neck squamous cell carcinoma (HNSC, HR:1.4; 95% CI; Logrank-p = 0.01), kidney renal papillary cell carcinoma (KIRP, HR:2.4; 95% CI; Logrank-p = 0.0072), LIHC (HR:1.6; 95% CI; Logrank-p = 0.011), lung adenocarcinoma (LUAD, HR:1.4; 95% CI; Logrank-p = 0.019), and uterine carcinosarcoma (UCS, HR:2.2; 95% CI; Logrank-p = 0.025), while patients of kidney renal clear cell carcinoma (KIRC, HR:0.67; 95% CI; Logrank-p = 0.0095), brain lower grade glioma (LGG, HR:0.68; 95% CI; Logrank-p = 0.037), and THCA (HR:0.3; 95% CI; Logrank-p = 0.028) with higher LRP11 level had better OS (Figure 1B upper panel and Supplementary Table S1). When analyzing the DFS, high expression of LRP11 was associated with a worse prognosis in ACC (HR:1.9; 95% CI; Logrank-p = 0.049), CESC (HR:2.6; 95% CI; Logrank-p = 0.0024), and LIHC (HR:1.9; 95% CI; Logrank-p<0.0001), while low expression of LRP11 indicated better prognosis in KIRC (HR:0.62; 95% CI; Logrank-p = 0.01) (Figure 1B lower panel and Supplementary Table S1). Because LRP11 expression was high and the prognosis was poor in ACC, CESC, and LIHC, LRP11 expression levels were further compared using TCGA and GTEX normal tissues. Here, the data demonstrated that LRP11 was significantly increased in LIHC tissues compared to that in normal liver tissues, while there was no change in ACC and CESC (Figure 1C). Thus, LRP11 is deemed a major prognostic factor for LIHC in various human cancers, and further studies have focused on LIHC.

### Clinical signification and validation analysis of the LRP11 in LIHC

**FIGURE 2:**
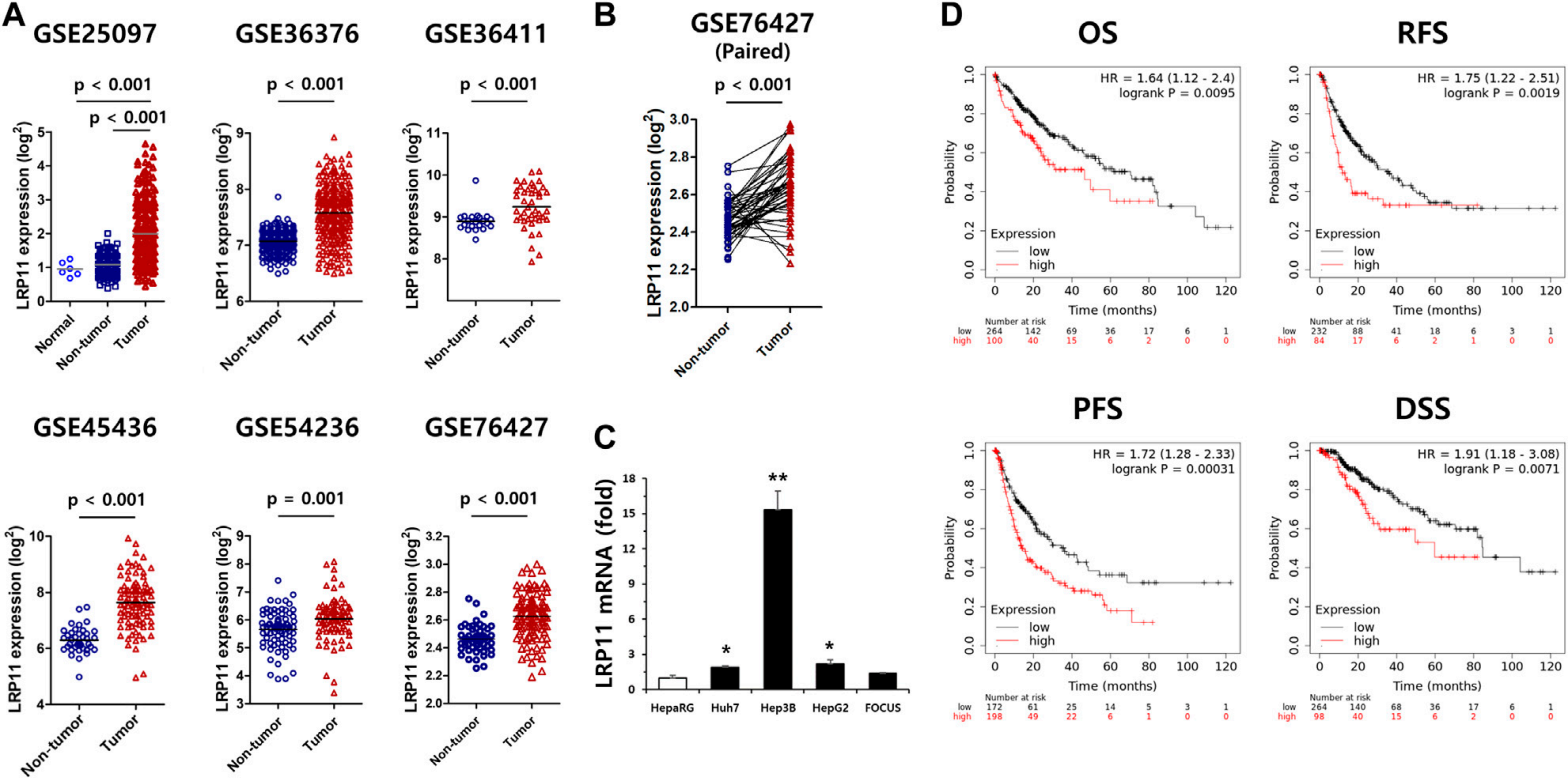
Validation of LRP11 expression and prognostic value of LRP11 in LIHC. **(A)** Validation of LRP11 expression in cohorts from the independent GEO dataset including GSE25097 (normal n = 5, non-tumor = 243, tumor = 268), GSE36376 (non-tumor n = 193, tumor = 240), GSE36411 (non-tumor n = 21, tumor = 42), GSE45436 (non-tumor n = 39, tumor n = 95), GSE54236 (non-tumor n = 80, tumor = 81), and GSE76427 (non-tumor n = 52, tumor n = 115). **(B)** The expression level of LRP11 in paired non-tumor (n = 52) and tumor tissues (n = 52) from the GSE76427 dataset. **(C)** Upregulation of LRP11 in LIHC cell lines. qRT-PCR were performed in 5 different liver cell lines. Data represent means ± s.e.m. *: vs. HepaRG. *p < 0.05 and **p < 0.01. **(D)** The prognostic value of the expression of LRP11 in Kaplan-Meier plotter: overall survival (OS), relapse-free survival (RFS), progression-free survival (PFS), and disease-specific survival (DSS). LRP11 expression levels; *p*-values derived from the log-rank test are indicated in each comparison and the " best cutoff " for LRP11 mRNA was used.

Because the expression of the LRP11 gene was upregulated in various cancers and was associated with the worst prognosis in LIHC, validation was performed on an additional six independent LIHC GEO databases. As shown in Figure 2A, the expression of LRP11 in tumors was significantly higher than that in non-tumor tissues and was considerably upregulated in the 52 LIHC-paired tumors (Figure 2B). In agreement with the results from the GEO database, LRP11 mRNA levels in LIHC cell lines were significantly upregulated compared to those in HepaRG cells, similar to human hepatocytes (Figure 2C). To better understand the role of LRP11 as a prognostic biomarker in LIHC, we used a Kaplan–Meier plot (KM-plot) to analyze the effect of LRP11 on survival time in LIHC. As expected, the high LRP11 group exhibited significantly worse OS, RFS, PFS, and DSS (Figure 2D). These results implied that LRP11 may play an oncogenic role in LIHC progression.

### Relationship between LRP11 expression and clinicopathological features in LIHC patients

**FIGURE 3:**
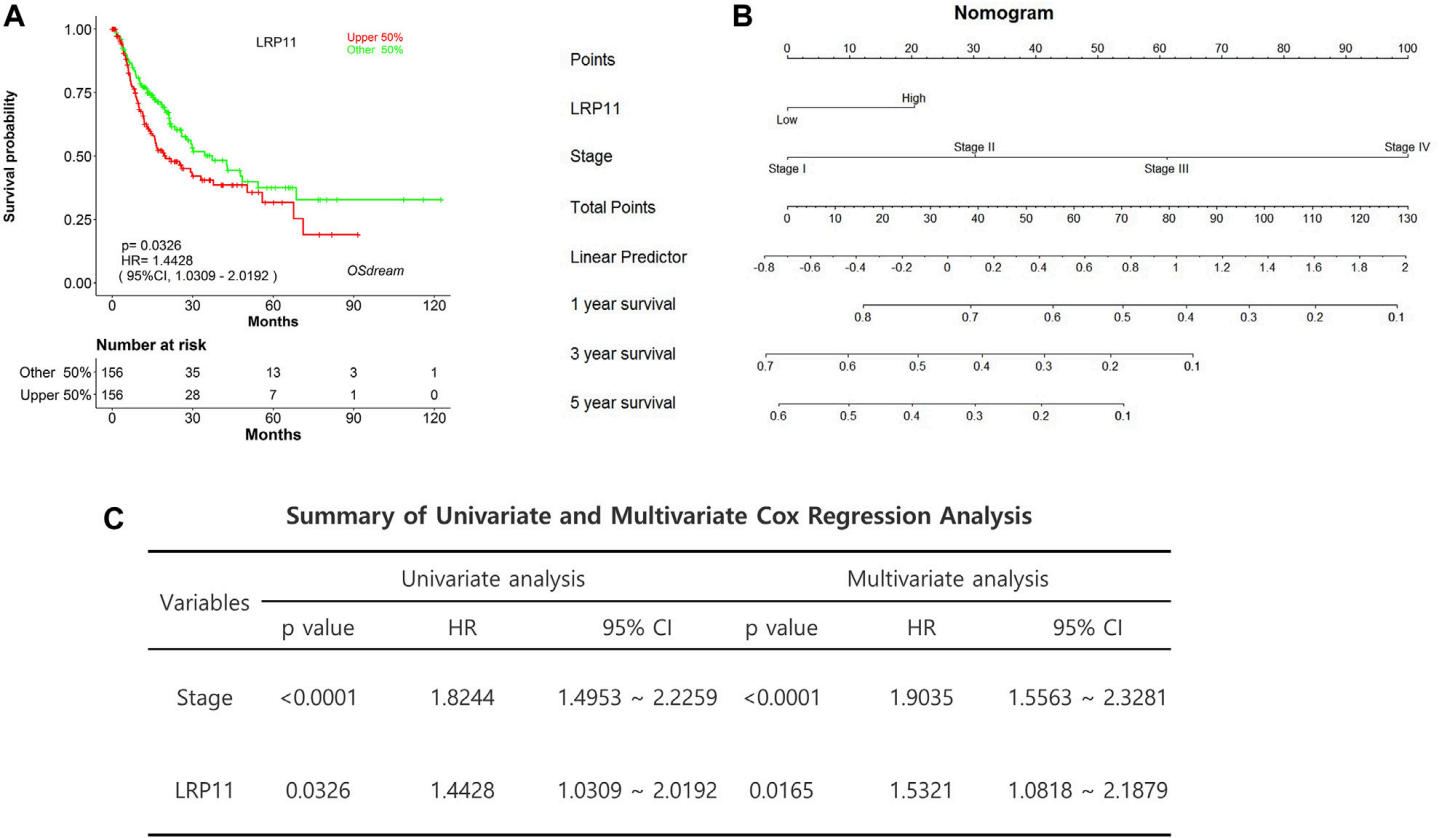
Cox regression analysis and nomogram predicting recurrence-free survival in LIHC patients. **(A)** Recurrence-free survival plot based on TCGA data from OSdream database. **(B)** Construction and verification of nomogram for predicting 1-, 3-, and 5-year survival in LIHC patients based on LRP11 expression. **(C)** Univariate and multivariate Cox regression analysis of LRP11.

We then determined the relationship between LRP11 expression and clinicopathological features of LIHC, including sex, age, race, tumor stage, tumor grade, and histological subtype, using TCGA data from the UALCAN database (Table 1). LRP11 expression was upregulated in different subgroups of LIHC compared to its expression in the corresponding normal group, demonstrating that LRP11 might be a potential diagnostic marker for LIHC patients. To enhance the model’s predictive power, we constructed a nomogram to predict recurrence-free survival using TCGA data from the OSdream database of LIHC patients. Kaplan-Meier analysis indicated that high LRP11 levels were associated with decreased recurrence-free survival (HR = 1.4428; *p* = 0.0326) (Figure 3A). According to the nomogram, the stage had the greatest influence on prognosis, and the total points were used to predict the probability of 1-, 3-, and 5-year survival, as displayed at the bottom of the nomogram (Figure 3B). Univariate and multivariate Cox regression analyses used the Cox proportional hazards model (Figure 3C). Univariate Cox analysis suggested that the increased mortality risk of LIHC patients was due to the clinical stage (HR:1.8244; 95% CI, 1.4953–2.2259; *p* < 0.0001) and LRP11 (HR:1.4428; 95% CI, 1.0309–2.0192; *p* = 0.0326). Multivariate Cox regression analysis also revealed that clinical stage clinical stage (HR:1.9035; 95% CI, 1.5563–2.3281; *p* < 0.0001) and LRP11 expression (HR:1.5321; 95% CI, 1.0818–2.1879; *p* = 0.0165). These results suggest that LRP11 is significantly correlated with clinical parameters in LIHC patients and may also be a promising biomarker for the postoperative management of patients.

**TABLE 1:** Clinicopathological features using TCGA data in HCC.

	Expression of LRP11 (transcript per million), median value	*p*-value
Sex (n = 412)		
Normal (n = 50)	1.709–6.712 (3.837)	
Male (n = 245)	1.211–26.723 (7.892)	<0.001*
Female (n = 117)	0.922–24.561 (9.692)	<0.001*
Age (n = 408)		
Normal (n = 50)	1.709–6.712 (3.837)	
Tumor (21–40, n = 27)	2.082–34.278 (10.494)	<0.001
Tumor (41–60, n = 140)	1.268–24.872 (8.694)	<0.001*
Tumor (61–80, n = 181)	1.211–24.561 (8.045)	<0.001*
Tumor (>80, n = 10)	0.922–18.748 (8.205)	0.017*
Race (n = 401)		
Normal (n = 50)	1.709–6.712 (3.837)	
Caucasian (n = 177)	0.922–23.099 (8.001)	<0.001*
African-american (n = 17)	3.208–29.826 (13.679)	<0.001*
Asian (n = 157)	1.268–28.254 (8.445)	<0.001*
Tumor stage (n = 390)		
Normal (n = 50)	1.709–6.712 (3.837)	
Stage I (n = 168)	0.922–24.872 (7.965)	<0.001*
Stage II (n = 84)	1.268–22.677 (8.078)	<0.001*
Stage III (n = 82)	1.211–32.666 (10.88)	<0.001*
Stage IV (n = 6)	5.974–12.662 (9.686)	n.s
Tumor grade (n = 407)		
Normal (n = 50)	1.709–6.712 (3.837)	
Grade I (n = 54)	1.487–19.807 (7.527)	<0.001*
Grade II (n = 173)	0.922–12.099 (8.009)	<0.001*
Grade III (n = 118)	1.619–30.386 (10.86)	<0.001*
Grade IV (n = 12)	2.006–16.232 (9.778)	0.042*
Histological subtype (n = 421)		
Normal (n = 50)	1.709–6.712 (3.831)	
Hepatocellular carcinoma (n = 361)	0.922–26.282 (8.421)	<0.001*
Fibrolamellar carcinoma (n = 3)	5.99–28.254 (13.058)	0.006*
Hepatocholangiocarcinoma (Mixed) (n = 7)	4.164–10.494 (4.923)	n.s
Nordal metastasis subtype (n = 421)		
Normal (n = 50)	1.709–6.712 (3.831)	
No regional lymph node metastasis (n = 252)	0.922–24.722 (8.699)	<0.001*
Metastases in 1–3 axillary lymph nodes (n = 4)	4.164–30.425 (20.396)	n.s

Notes: * vs normal.

### Association between LRP11 expression and tumor microenvironment (TME) in LIHC

**FIGURE 4:**
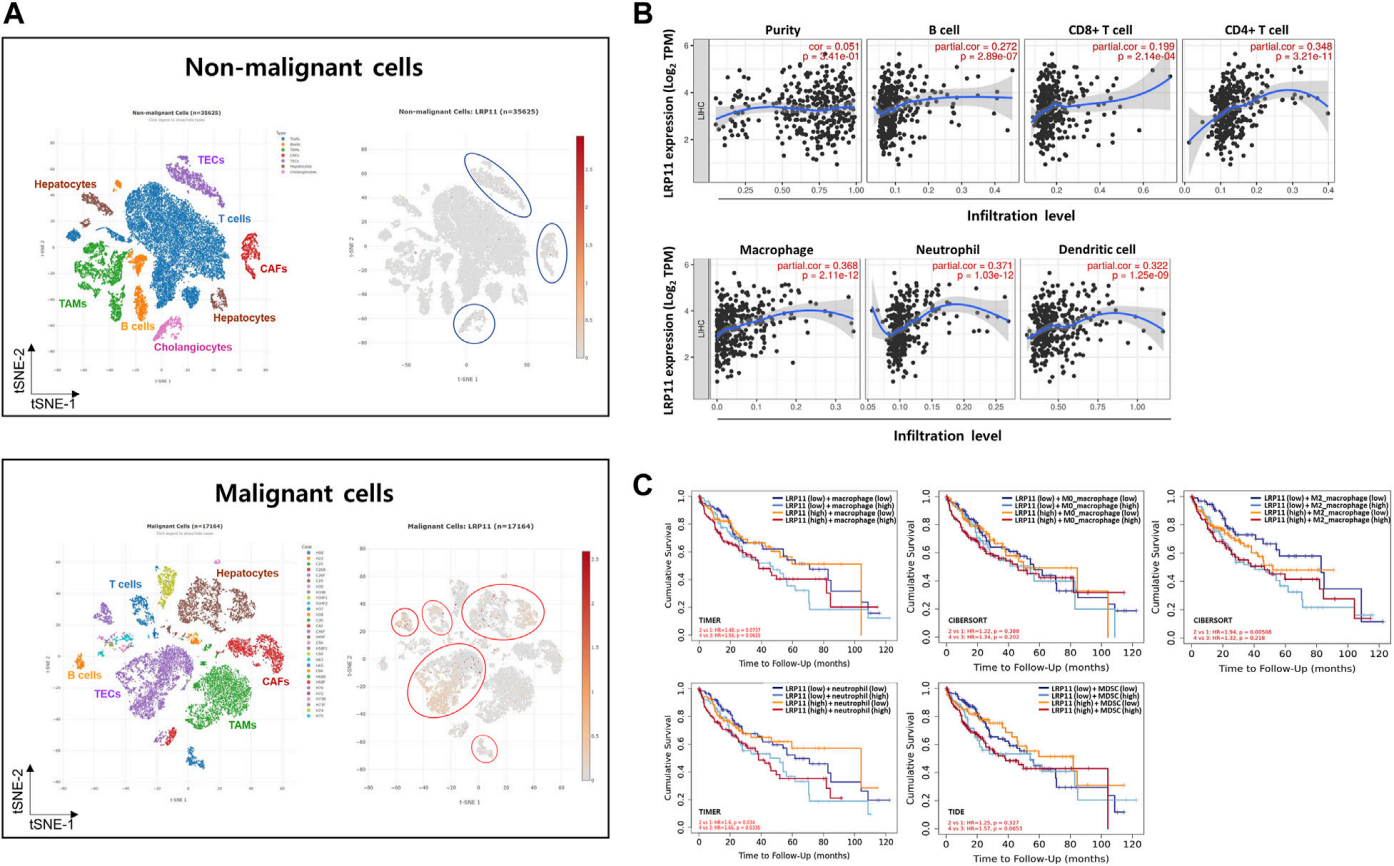
Characterization of LRP11 expression on TME in LIHC. Single-cell analysis of LRP11 gene using scAtlasLC **(A)**. **(B)** Correlation analysis between LRP11 expression and immune infiltrates in LIHC. **(C)** Comprehensive prognostic value of LRP11 expression and macrophage, macrophage M0, macrophage M2, neutrophile, and MDSC infiltration levels based on the TIMER algorithm. CAFs, cancer-associated fibroblasts; TAMs, tumor associated macrophages; TECs, tumor-associated endothelial cells.

**FIGURE 5:**
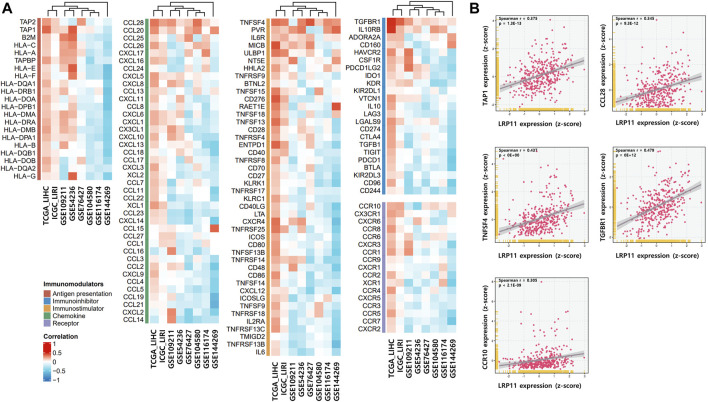
Correlation between LRP11 expression and immunomodulatory genes. **(A)** The correlation between LRP11 expression and immunomodulator. **(B)** Diagrams of Spearman’s correlation between LRP11 (TCGA-LIHC) and representative immunomodulator genes.

To explore the potential molecular mechanism of LRP11 in LIHC, we investigated the association between LRP11 expression and TME profiling using scAtlasLC datasets. LRP11 was mainly expressed in hepatocytes, T cells, and tumor-associated endothelial cells (TECs) in malignant cells. In contrast, it was weakly expressed in TECs, cholangiocytes, and cancer-associated fibroblasts in nonmalignant cells (Figure 4A). Next, we analyzed LRP11 expression and immune cell infiltration in LIHC cells using TIMER. As shown in Figure 4B, the expression of LRP11 significantly correlated with B cells (r = 0.272, *p* = 2.89e-07), CD8^+^ T cells (r = 0.199, *p* = 2.14e-04), CD4^+^ T cells (r = 0.348, *p* = 3.21e-11), macrophages (r = 0.368, *p* = 2.11e-12), neutrophils (r = 0.371, *p* = 1.03e-12), and dendritic cells (r = 0.322, *p* = 1.25e-09) in LIHC. In addition, a correlation analysis was performed between LRP11 expression and infiltrating immune cells in LIHC using the TIMER and GEPIA databases. As shown in Table 2, LRP11 expression positively correlated with B cell (CD17 and CD79A), T cell general (CD3E, CD2), CD8^+^ T cell (CD8A), CD4^+^ T cell (CD4), TAM (CD68, IL10), M1 macrophages (NOS2, IRF5, and PTGS2), M2 macrophages (VSIG4, MS4A4A), neutrophils (ITGAM,CCR7), natural killer cell (KIR2DL4, KIR2DL3, KIR3DL3, KIR3DL2), DC (HLA-DPB1, HLA-DRA, HLA-DPA1, NRP1, ITGAX), Th1 (STAT4, STAT1, IFNG, TNF), Th2 (GATA3, STAT6, IL13, STAT5A), Tfh (BCL6), Th17 (STAT3), Treg (FOXP3, CCR8, TGFB1, STAT5B), and T cell exhaustion (PDCD1, CTLA4, LAG3, HAVCR2). To further expand and correlate the results of immune cell infiltration, a comprehensive prognostic analysis was performed to compare LRP11 expression and infiltrating immune cells in LIHC. The results revealed that the low expression of LRP11 and low immune infiltration of macrophages, macrophage M0, macrophage M2, and myeloid-derived suppressor cells (MDSC) was associated with better prognosis than that associated with a high expression of LRP11 in LIHC (Figure 4C). At the same time, no significant correlation was observed in CD8^+^ T cells, CD4^+^ T cells, B cells, neutrophils, DC, and NK cells (data not shown). We further analyzed the role of LRP11 in immunomodulation. Through difference analysis and correlation analysis, we found that LRP 11 was positively associated with many immunomodulators including TAP1, TAP2, and B2M, HLA class on antigen presentation; CCL28, CCL20, and CCL25 on chemokine, TNFSF4, PVR, and IL6R on immunostimulator; TGFBR1, IL10RB, and ADORA2A on immunoinhibitor; CCR10 on receptor (Figures 5A, B). These findings suggest that LRP11 expression is not only correlated with immune cell infiltration and immunomodulation, but also plays a role in the prognosis of LIHC.

**TABLE 2:** Correlation between LRP11 expression level and gene markers of tumor infiltrating immune cells in TCGA-LIHC.

Immune cell	Biomarker	R-value	*p*-value
B cell	CD19	0.22	***
	CD79A	0.13	*
T cell (general)	CD3D	0.097	n.s
	CD3E	0.14	**
	CD2	0.14	**
CD8^+^ T cell	CD8A	0.13	*
	CD8B	0.059	n.s
CD4^+^ T cell	CD4	0.2	***
TAM	CCL3	0.01	n.s
	CD68	0.18	***
	IL10	0.23	***
M1 macrophage	NOS2	0.2	***
	IRF5	0.47	***
	PTGS2	0.25	***
M2 macrophage	CD163	0.021	n.s
	VSIG4	0.15	**
	MS4A4A	0.15	**
Neutrophil	CEACAM8	0.019	n.s
	ITGAM	0.34	***
	CCR7	0.11	*
Natural killer cell	KIR2DL4	0.13	*
	KIR2DL3	0.13	*
	KIR3DL3	0.11	*
	KIR3DL2	0.18	***
	KIR2DS4	0.023	n.s
	KIR2DL1	0.084	n.s
	KIR3DL1	0.029	n.s
Dendritic cell	HLA-DPB1	0.15	**
	HLA-DQB1	0.036	n.s
	HLA-DRA	0.2	***
	HLA-DPA1	0.19	***
	CD1C	0.093	n.s
	NRP1	0.27	***
	ITGAX	0.3	***
Th1	TBX21	0.087	n.s
	STAT4	0.26	***
	STAT1	0.38	***
	IFNG	0.12	*
	TNF	0.2	***
Th2	GATA3	0.21	***
	STAT6	0.33	***
	IL13	0.17	**
	STAT5A	0.23	***
Tfh	BCL6	0.25	***
	IL21	0.068	n.s
Th17	STAT3	0.33	***
	IL17A	0.063	n.s
Treg	FOXP3	0.18	***
	CCR8	0.34	***
	TGFB1	0.18	***
	STAT5B	0.38	***
T cell exhaustion	PDCD1	0.17	***
	CTLA4	0.21	***
	LAG3	0.14	**
	HAVCR2	0.26	***
	GZMB	−0.06	n.s

Notes: *p < 0.05; **p < 0.01; ***p < 0.001.

### Association between LRP11 gene methylation and clinicopathological features of LIHC patients

**FIGURE 6:**
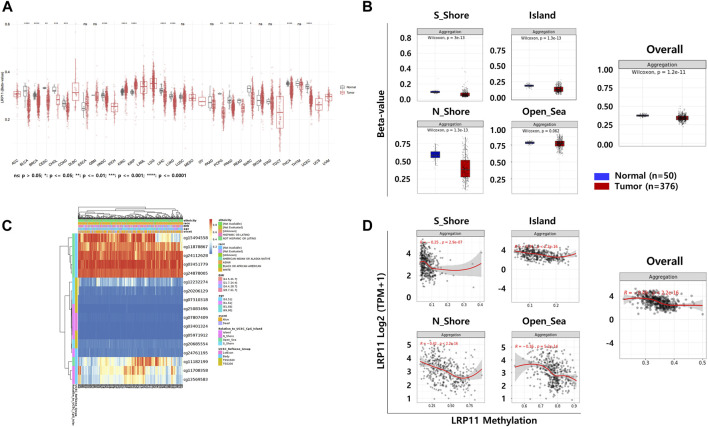
The DNA methylation of LRP11 in LIHC from TCGA data. **(A)** The methylation levels of LRP11 across tumor tissues and corresponding normal tissues in SMART database. **(B)** Heatmap integrating DNA methylation of the LRP11 gene in LIHC by MethSurv. **(C)** Average methylation levels between normal and tumor tissue stratified by genomic location (Wilcoxon rank sum test). **(D)** Spearman’s correlation between mRNA expression of LRP11 and methylation level. S_Shore, South Shore; N_Shore, North Shore.

**FIGURE 7:**
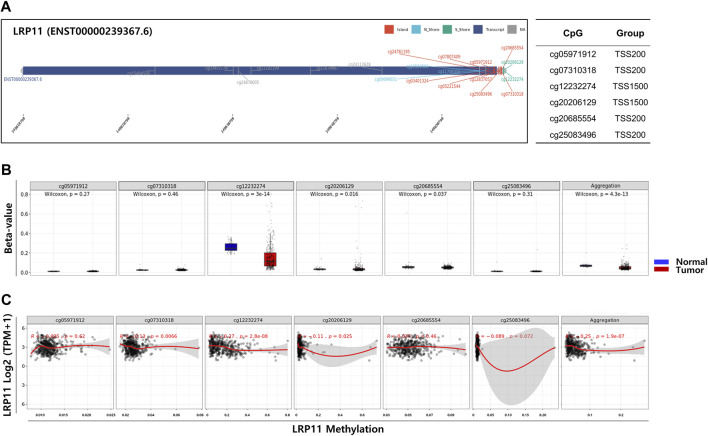
Correlation analysis between DNA methylation and gene expression in LRP11 promoter region. **(A)** Chromosomal distribution and detailed CpG sites. The promoter region includes six probes (cg05971912, TSS200; cg7310318, TSS200; cg12232274, TSS1500; cg20206129, TSS1500; cg20685554, TSS200; cg25083496, TSS200). **(B)** The methylation level of six probes between LIHC tumor group and normal group. **(C)** Spearman’s correlation between LRP11 expression and methylated sites.

To explore the potential mechanism of LRP11 upregulation in LIHC, we analyzed the methylation levels of LRP11 using the SMART database. As shown in Figure 6A, the methylation levels of LRP11 were significantly lower in LIHC tissues than in normal liver tissues. Specific methylation positions are illustrated on heatmaps using the MethSurv database (Figure 6B). In total, we found that the average methylation of all 17 CpG sites on LRP11 (Overall; Aggregation, *p* = 2.5e-10), including S_Shore (Aggregation, p = 3e-13), Island (Aggregation, *p* = 1.3e-13), and N_Shore (Aggregation, *p* = 1.3e-13), was significantly lower in LIHC tissues than in normal liver tissues. At the same time, Open_Sea was not significantly changed (Aggregation, *p* = 0.062) (Figure 6C and Supplementary Figure S1). In the correlation analysis between the methylation of LRP11 and LRP11 expression, we found a negative correlation between the methylation level and the expression of LRP11 (Overall; Aggregation, *p* = 2.2e-16), including S_Shore (aggregation, *p* = 2.9e-07), Island (aggregation, *p* = 2.2e-16), N_Shore (aggregation, *p* = 2.2e-16), and Open_Sea (aggregation, *p* = 5.2e-14) (Figure 6D and Supplementary Figure S2). Based on the above methylation profile, we determined whether the cause of the increased expression of LRP11 was hypomethylation in the promoter region (Figure 7A). Notably, we found that the average methylation level of CpGs on the predominant form of the LRP11 promoter was significantly downregulated in the tumor group compared to its expression in the corresponding normal group (aggregation, *p* = 4.3e-13) (Figure 7B) and negatively correlated with the expression levels of LRP11 (aggregation, *p* = 1.9e-07) (Figure 7C), suggesting that the increase in LRP11 expression might be tightly regulated by promoter methylation. Considering the previously mentioned results, we assessed the correlation between methylation and the prognosis of the LRP11 gene in LIHC. As shown in Table 3, cg1549455 (HR = 0.7; *p* = 0.0423), cg11708358 (HR = 0.71; *p* = 0.0487), and cg12232274 (HR = 0.7; *p* = 0.0415) predicted better prognosis of LIHC patients, while cg24112628 (HR = 1.47; *p* = 0.0273), cg07807409 (HR = 1.8; *p* = 0.0008), and cg25083496 (HR = 1.47; *p* = 0.0273) were associated with poor clinical outcomes. Collectively, these results suggest a mechanism by which the expression of LRP11 could be regulated by promoter methylation but also suggest that methylation profiling might be a prognostic biomarker in LIHC patients.

**TABLE 3:** CpGs methylation across the patients with Hepatocellular carcinoma based on SMART database.

CGIposition	UCSC_RefGene_Group	Probe	Overall survival	
			Hazard ratio (95% CI) cutoff-high 50%, -low 50%	*p*-value
Open_Sea	Body	cg1549455g	0.7 (0.5–0.99)	0.0423
	Body	cg03451779	0.89 (0.63–1.25)	n.s
	Body	cg24878005	0.92 (0.64–1.27)	n.s
	Body	cg11182199	0.91 (0.64–1.27)	n.s
	Body	cg11878867	0.86 (0.61–1.21)	n.s
	Body	cg24112628	1.47 (1.05–2.07)	0.0273
N_Shore	Body	cg11708358	0.71 (0.51–1)	0.0487
	Body	cg13569583	0.89 (0.63–1.25)	n.s
Island	Body	cg24761195	1.07 (0.76–1.51)	n.s
	1stExon	cg03401324	1.02 (0.72–1.43)	n.s
	1stExon	cg07807409	1.8 (1.28–2.53)	0.0008
	TSS200	cg05971912	1.2 (0.86–1.69)	n.s
	TSS200	cg25083496	1.47 (1.04–2.07)	0.0273
	TSS200	cg07310318	0.81 (0.58–1.14)	n.s
	TSS200	cg20685554	1.06 (0.76–1.49)	n.s
S_Shore	TSS1500	cg20206129	0.93 (0.66–1.3)	n.s
	TSS1500	cg12232274	0.7 (0.5–0.99)	0.0415

Note: UCSC_RefGene_Group (gene region, based on UCSC, annotations).

### Biological analyses and target drug prediction of LRP11 in LIHC

**FIGURE 8:**
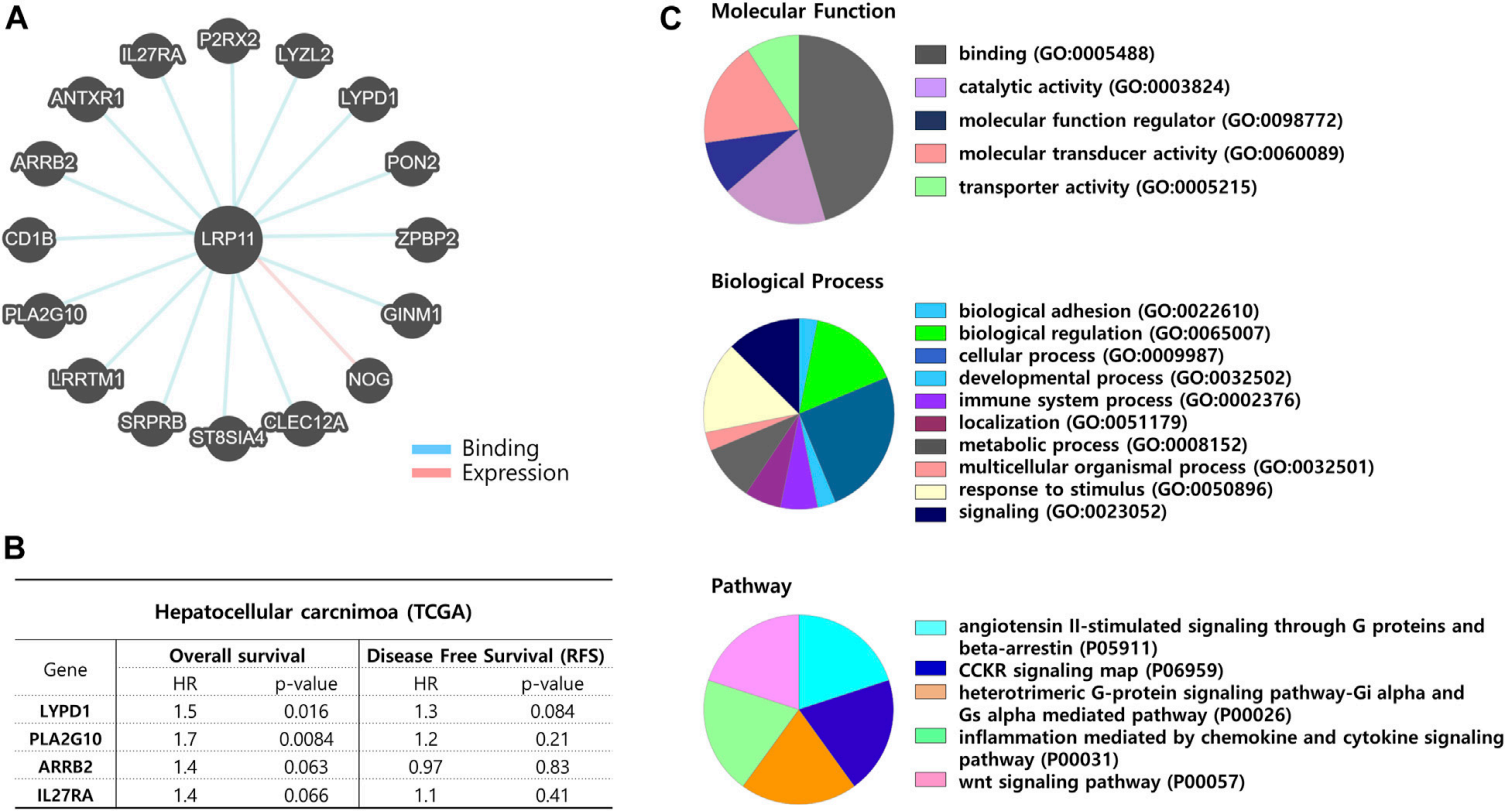
Network analysis between LRP11 and interacting proteins. **(A)** Diagram of potential interacted proteins with LRP11 by Pathways Commons. **(B)** Interacting protein list of statistically significant between prognosis and LRP11 expression in LIHC-TCGA. **(C)** Pie charts showing the molecular function, biological process, and pathway class from PANTHER database.

**FIGURE 9:**
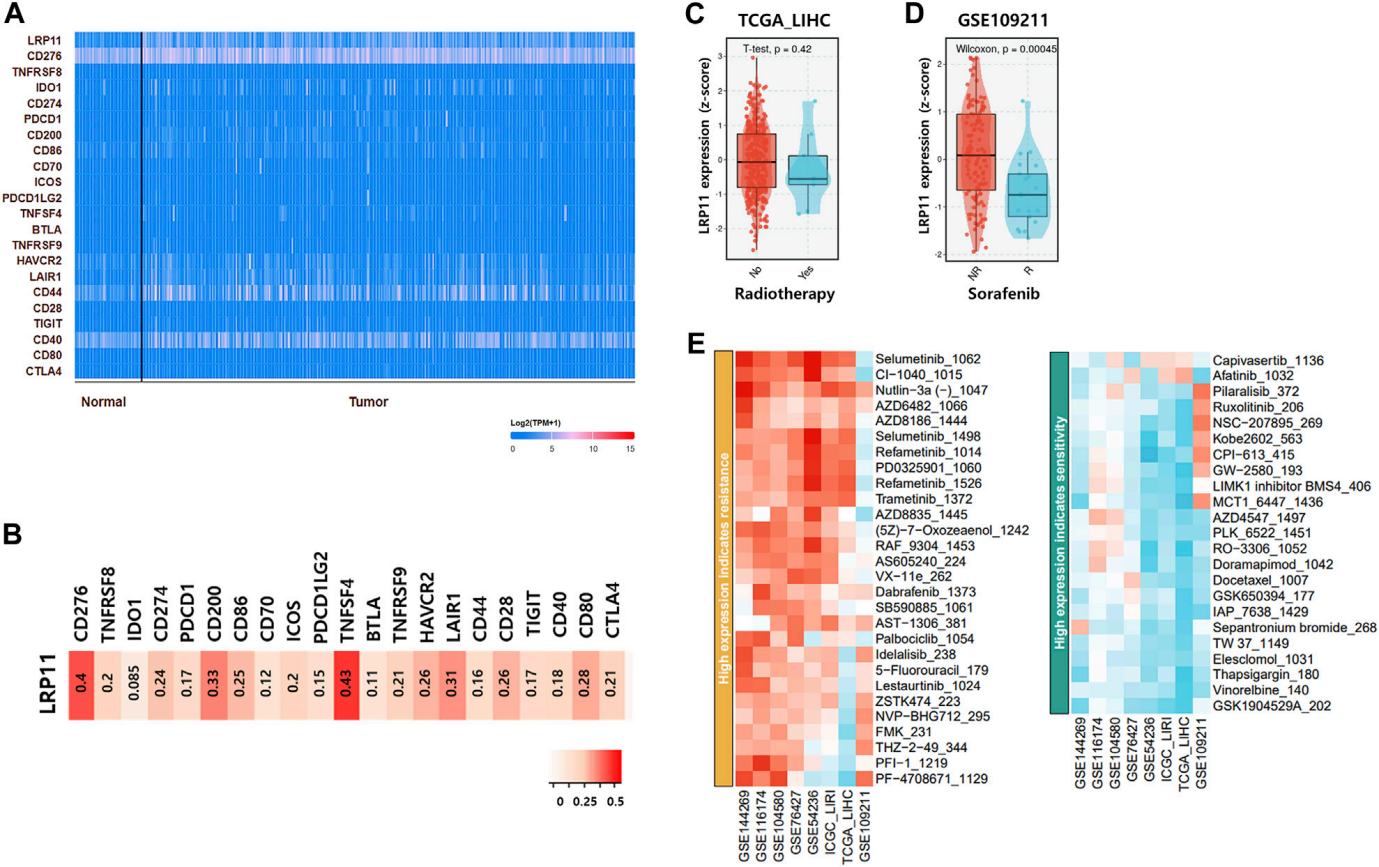
Effect of LRP11 on ICI-related gene efficacy and in LIHC patients. **(A)** Schematic of the 24 gene expression clusters containing LRP11 defined in TCGA-LIHC. **(B)** Spearman’s correlation analysis of LRP11 across the 23 ICI-related gene clusters. **(C)** LRP11 expression in radiotherapy group and no-radiotherapy treatment group based on TCGA-LIHC. **(D)** Comparison of LRP11 in sorafenib responders and nonresponders based on GSE109211. **(E)** Heatmap integrating candidate agents of LRP11 in LIHC by Genomics of Drug Sensitivity in Cancer (GDSC1) database.

Next, to verify the biological role of LRP11, genes interacting with LRP11 were identified using Pathway Commons, and a prognostic analysis was conducted in the normal and LIHC groups (Figure 8A; Table 4). Based on the prognostic analysis, LIHC-specific LRP11-related proteins were selected (Figure 8B), and these proteins were further used for subsequent bioinformatic analyses according to the PANTHER database regarding functional clusters. As shown in Figure 8C, the functions of LRP11 and its interacting proteins were classified into molecular functions, biological processes, and pathways. The results demonstrated that proteins were mainly enriched in molecular function including “binding”, “catalytic activity”, and “molecular transducer activity”; in biological processes including “cellular process”, “biological regulation”, and “response to stimulus”; in pathway including “angiotensin II-stimulated signaling through G proteins and beta-arrestin”, “CCKR signaling map”, “heterotrimeric G-protein signaling pathway-Gi alpha and Gs alpha mediated pathway”, “inflammation mediated by chemokine and cytokine signaling pathway”, and “Wnt signaling pathway”. Because the therapeutic efficacy of immune checkpoint inhibitors is associated with the expression of target genes and their complementary receptors, the expression of LRP11 and 23 ICI-related genes was evaluated based on gene expression data from TCGA-LIHC (Figure 9A). The results revealed that the expression of CD276, CD274, CD200, CD86, TNFSF4, HAVCR2, LAIR1, CD28, and CD80 positively correlated with LRP11 expression in LIHC (Figure 9B). Furthermore, when analyzing the relationships between standard treatment for LIHC and LRP11 expression, we found that among all the patients in sorafenib treatment of GSE109211 rather than radiotherapy treated group in TCGA-LIHC, the patients in the non-responding group showed relatively upregulated in LRP11 expression (Figures 9C, D). In addition, elevated LRP11 expression was positively correlated with resistance to various chemotherapy drugs, including Selumetinib, Nutlin-3a, and AZD6482, while it was associated with sensitivity including GSK1904529A, Thapsigargin, and Elesclomol in LIHC patients (Figure 9E). These results indicate that LRP11 may play a biological role in LIHC through these pathways, and LRP11-specifically related genes could also be associated with poor prognosis and affect the efficacy of ICIs and chemotherapeutics.

**TABLE 4:** Correlation analysis between LRP11 and predicted interactions in pathways commons.

	Normal				HCC			
	Pearson correlation	*p*-value	Spearman correlation	*p*-value	Pearson correlation	*p*-value	Spearman correlation	*p*-value
P2RX2	−0.044	n.s	0.32	0.024	−0.067	n.s	0.072	n.s
LYZL2	−0.008	n.s	0.015	n.s	0.016	n.s	−0.054	n.s
**LYPD1**	0.2	n.s	0.25	n.s	**0.11**	**0.038**	**0.23**	**<0.001**
PON2	0.72	<0.001	0.61	<0.001	0.18	<0.001	0.24	<0.001
ZPBP2	0.21	n.s	0.13	n.s	0.014	n.s	−0.002	n.s
GINM1	0.64	<0.001	0.5	<0.001	0.55	<0.001	0.54	<0.001
NOG	−0.083	n.s	0.12	n.s	0.086	n.s	0.086	n.s
CLEC12A	0.31	0.027	0.41	0.034	0.11	0.036	0.15	0.005
ST8SIA4	0.43	0.002	0.53	<0.001	0.25	<0.001	0.35	<0.001
SRPRB	0.65	<0.001	0.55	<0.001	0.17	0.001	0.13	0.01
LRRTM1	0.26	n.s	0.27	n.s	−0.076	n.s	−0.024	n.s
**PLA2G10**	0.31	0.03	0.26	n.s	**0.21**	**<0.001**	**0.3**	**<0.001**
CD1B	0.3	0.034	0.34	0.017	0.096	n.s	0.15	0.003
**ARRB2**	0.17	n.s	0.35	0.013	**0.33**	**<0.001**	**0.39**	**<0.001**
ANTXR1	0.52	<0.001	0.62	<0.001	0.21	<0.001	0.29	<0.001
**IL27RA**	0.25	n.s	0.36	0.01	**0.3**	**<0.001**	**0.34**	**<0.001**

Bold indicates statistical significance in HCC group only.

### The effects of LRP11 on malignant phenotype of hepatocellular carcinoma cells

**FIGURE 10:**
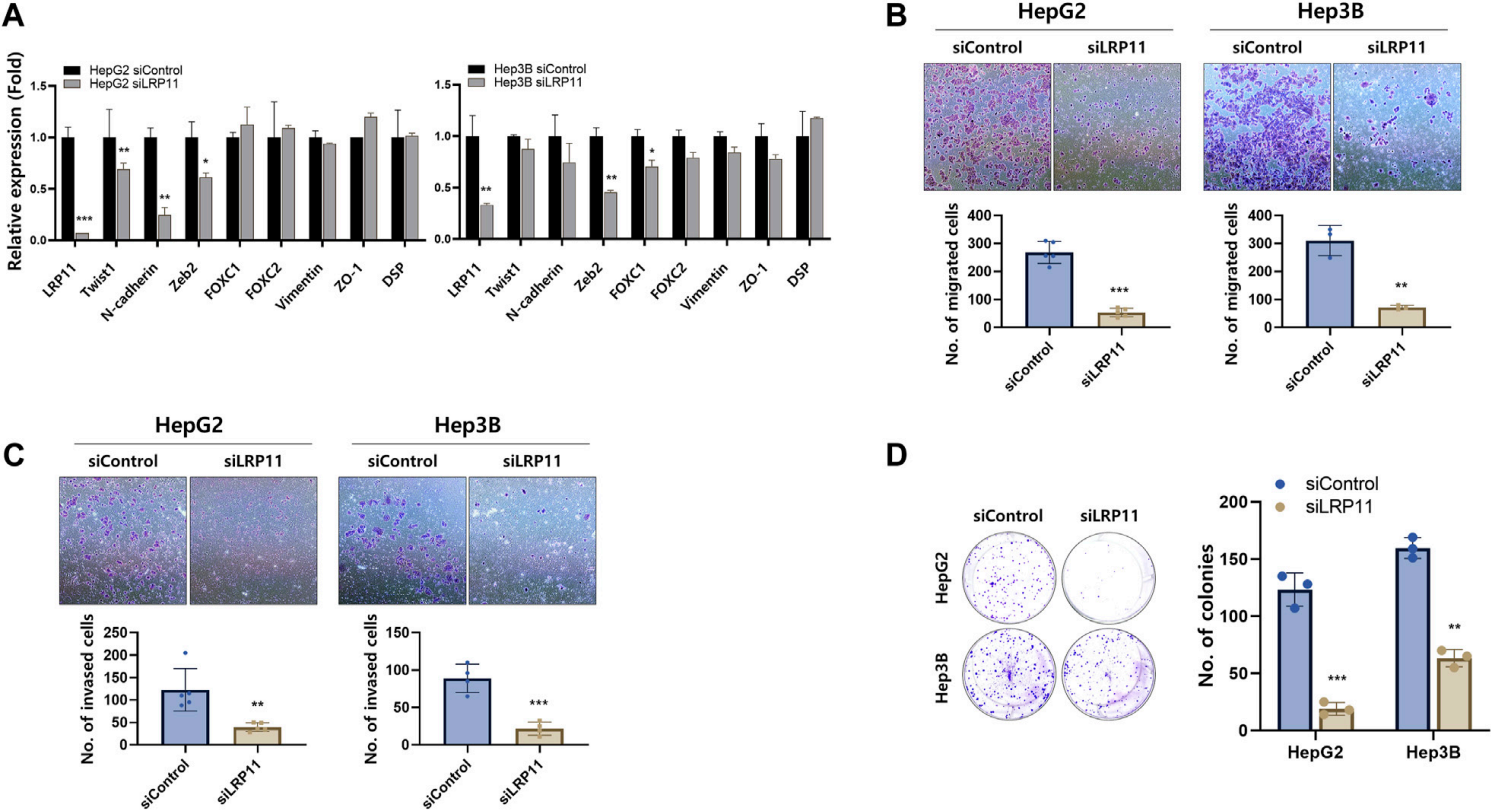
Effect of LRP11 on malignant phenotype of HepG2 and Hep3B cells. **(A)** qRT-PCR analysis of EMT and MET marker expression in HepG2 and Hep3B cells transfected with siControl or siLRP11 transfection. **(B)** Migration and **(C)** invasion ability assessed by transwell assay (magnification, ×100). **(D)** Colony formation ability and quantification of colonies showed cell growth of the indicated cells (magnification, ×100). Data represent means ± s.e.m. *: vs. siControl. *p < 0.05, **p < 0.01, and ***p < 0.001.

Based on previous findings of LRP11 expression in various LIHC cell lines (Figure 2C), HepG2 and Hep3B cells were selected, and these cell lines were further validated the malignant performance of LRP11. Interestingly, siRNA-LRP11 decreased levels of EMT markers, including Twist1, N-cadherin, and Zeb2 in HepG2 cells, and Zeb2 and FOXC1 in Hep3B cells; however, the expression of mesenchymal epithelial transition markers (MET) including ZO-1 and DPS was not changed in these 2 cells (Figure 10A). Cell migration and invasion depend on EMT; therefore, we next performed transwell migration and invasion assay. After downregulating LRP11 in HepG2 and Hep3B cells, their ability of migration and invasion was significantly lower than those of control group (Figure 10C). Moreover, the result of colony formation assay showed that knockdown of LRP11 significantly inhibited colony-forming efficiency (Figure 10D). Taken together, these results demonstrated that LRP11 enhanced the malignant ability of LIHC cells.

## Discussion

Hepatocellular carcinoma is one of the deadliest malignant tumors worldwide because of its intratumor, intrapatient, and interpatient heterogeneity (Chan et al., 2022). The prognosis of LIHC is mainly determined at the tumor stage, and the survival rate for advanced-stage patients undergoing systemic treatment is approximately 1–1.5 years. In contrast, the 5-year survival rate exceeds 70% for patients with early diagnosed LIHC (Llovet et al., 2016; Villanueva, 2019). Although various treatments are available for LIHC, the survival rate remains unsatisfactory. Therefore, there is an urgent need to identify additional biomarkers to contribute new insights into treatment decision management through a biological understanding of LIHC.

In our study, we analyzed data from the TCGA database and found that LRP11 was upregulated and was associated with poor survival in LIHC. Although the prognostic value of LRP11 has been previously reported in prostate and cervical cancers, its role of LRP11 in LIHC remains unclear. To our knowledge, this study of LRP11 in LIHC is the first to comprehensively evaluate the prognostic value, tumor microenvironment, methylation profiling, gene networks, and biological functions. In the present study, results from various databases indicated that LRP11 was upregulated in multiple cancers, including LIHC, and that patients with adverse clinicopathological characteristics had high levels of LRP11 expression. Kaplan–Meier survival analysis of OS, RFS, PFS, and DSS revealed that LIHC patients with a higher level of LRP11 had a shorter survival time, consistent with the nomogram validation showing dependency on the LIHC stage in recurrence-free survival. Subsequently, we validated knockdown of LRP11 markedly reduced the capacity of cell migration, invasion, and colony formation of LIHC cells, at least partly dependent on EMT. These findings strongly suggest that high LRP11 expression is closely associated with worse outcomes and might be involved in malignancy of LIHC patients.

The tumor microenvironment consists of stromal cells, fibroblasts, endothelial cells, and immune cells and plays a key role in tumor development (Sadeghi Rad et al., 2021). Because these cells organize a microenvironment favorable for tumor progression *via* cell-to-cell interactions or the release of various molecules, this correlation could be an important factor in determining the effectiveness of cancer immunotherapy and is strongly associated with the prognosis of multiple cancers (Satge, 2018; Riley et al., 2019). This study found that LRP11 was mainly expressed in the T cells, hepatocytes, and TECs of malignant LIHC cells. Further correlation analysis revealed that the expression of LRP11 was positively associated with B cells, CD8^+^ T cells, CD4^+^ T cells, macrophages, neutrophils, and DC in LIHC. Moreover, we observed that high LRP11 expression and high infiltration of immune cells, including macrophages, neutrophils, and MDSC, were associated with the worst patient prognosis, emphasizing the importance of LRP11 and its role in cancer-related immune processes. Immunomodulators provide an opportunity to design powerful substances that modify or regulate the immune system to help the body respond to cancers, as well as the interaction between anti-cancer agents and cancer cells (Khurana et al., 2019). Therefore, understanding the immunomodulatory response provides a strong basis for developing combination therapy for cancers, including immunotherapy. We identified a correlation between LRP11 gene and immunomodulators in various LIHC datasets and demonstrated that the expression of TAP1, CCL28, TNFSF4, TGFBR1, and CCR10 was shown to be linked with positive association in the levels of LRP11 mRNA. In the previous bioinformatic analysis, the high expression of TAP1 and TNFSF4 were risk factors in patients with LIHC (Tabassum et al., 2021; Xu et al., 2023). According to Mazzocca et al. (2009), transforming growth factor beta (TGF-β) receptor 1 (TGFR1) is shown to involve in neo-angiogenesis and tumor growth in LIHC and it has been proved that CCL28 and CCR10 play a significant role in tumor growth and carcinogenesis in LIHC (Ren et al., 2016; Wu et al., 2018). The analysis of the present study revealed a significant correlation between LRP11 and immunomodulatory genes, especially carcinogenesis and prognosis in LIHC, so it is hypothesized that LRP11 is linked with the immune pattern of LIHC including immunomodulators, as well as the useful therapeutic targets.

Because DNA methylation is an important epigenetic factor for gene expression, we next analyzed LRP11 expression at the epigenetic level. Methylation profiling indicated that the overall methylation levels were low in LIHC, which positively correlated with LRP11 expression. Subsequently, hypomethylation of LRP11 in the promoter region significantly correlated with worse prognosis in patients with LIHC. Hypomethylation of the gene promoter tended to correlate with gene expression positively, and the inverse correlation between promoter methylation of LRP11 and its expression levels were consistent with previous studies (Herman and Baylin, 2003; Jones, 2012).

To further explore the biological function of LRP11, we selected four genes with prognostic power for LIHC among the 16 proteins that interacted with LRP11. Functional analysis revealed that LRP11-related genes were closely associated with multiple intracellular functions, such as regulation, transduction, responses to stimuli, and binding activity, in addition to some signaling pathways, including G-protein-mediated signaling and Wnt signaling. Notably, G-protein-coupled receptors and Wnt/β-catenin signaling were involved in pathophysiological mechanisms, including cancer progression, metastasis, and poor prognosis (Bagnato and Rosano, 2019; Yu et al., 2021). Thus, the functional analysis of LRP11 suggested that it may have a certain impact on tumor occurrence and prognosis and provide new insights into the recognition and challenges aimed at such signaling in cancer. Moreover, we found that the expression of CD276, CD274, CD200, CD86, TNFSF4, HAVCR2, LAIR1, CD28, and CD80 positively correlated with that of LRP11 in LIHC. These genes were well-characterized immune checkpoint biomarkers that reflect the effects of immunotherapy. Therefore, our findings indicated that LRP11 tended to respond effectively to immunotherapy, although it may promote the development and progression of LIHC. Through the correlation analysis between LRP11 expression and sorafenib, which has been considered the standard of patients with LIHC, we found that LRP11 was upregulated in sorafenib nonresponse group. Sorafenib was the only first-line drug approved by FDA for patients with advanced LIHC, however only 30% of LIHC patients are responding to Sorafenib. Based on the accumulating results of clinical trials and experimental evidences, it was confirmed that LIHC patients showed existing innate or acquired resistance to sorafenib (Llovet et al., 2008; Ford et al., 2009). In addition, we also observed LRP11 expression was significantly linked with drug sensitivities such as Selumetinib-, Nutlin-3a, and AZD6482 in GDSC database, this suggests that LRP11 is likely to mediate acquired or innate drug resistance to various drugs, as sorafenib response in LIHC patients.

## Conclusion

In the present study, bioinformatics analysis confirmed that LRP11 may be important for the development and prognosis of LIHC. However, because our study was conducted based on bioinformatics analysis, further clinical and experimental validation should be conducted to confirm the results of this prediction in LIHC. We hope the current research provided new insights to potentially be used as cancer treatment and prognostic biomarkers for LIHC.

## Data Availability

The original contributions presented in the study are included in the article/Supplementary Materials, further inquiries can be directed to the corresponding authors.
