# Supplementary material for: Comprehensive analysis on clinical significance and therapeutic targets of LDL receptor related protein 11 (LRP11) in liver hepatocellular carcinoma
Source: Front Pharmacol. 2024 Feb 15;15:1338929. doi: 10.3389/fphar.2024.1338929 (PMC10902445; doi:10.3389/fphar.2024.1338929)
Supplement: Supplementary file 1 [file Table2.DOCX]

**Supplementary Table 1.** Prognostic analysis of LRP11 gene in pan-cancer using GEPIA2 database

| **Tumor type** | **Overall survival (OS)** | | **Disease Free Survival (RFS)** | |
| --- | --- | --- | --- | --- |
|  | **HR (95% CI)** | **Logrank p** | **HR (95% CI)** | **Logrank p** |
| ACC | 2.5 | 0.02 | 1.9 | 0.049 |
| BLCA | 1.3 | 0.11 | 1.2 | 0.31 |
| BRCA | 1.7 | < 0.001 | 0.81 | 0.27 |
| CESC | 2 | 0.0049 | 2.6 | 0.0024 |
| CHOL | 0.85 | 0.74 | 0.71 | 0.48 |
| COAD | 1.3 | 0.3 | 1.1 | 0.62 |
| DLBC | 1.7 | 0.46 | 2.8 | 0.11 |
| ESCA | 1.2 | 0.36 | 1.1 | 0.69 |
| GBM | 1.1 | 0.5 | 1 | 0.91 |
| HNSC | 1.4 | 0.01 | 1.3 | 0.15 |
| KICH | 0.82 | 0.77 | 0.67 | 0.54 |
| KIRC | 0.67 | 0.0095 | 0.62 | 0.01 |
| KIRP | 2.4 | 0.0072 | 1.1 | 0.71 |
| LAML | 0.87 | 0.61 | 1 | 1 |
| LGG | 0.68 | 0.037 | 0.74 | 0.06 |
| LIHC | 1.6 | 0.011 | 1.9 | < 0.0001 |
| LUAD | 1.4 | 0.019 | 1.3 | 0.11 |
| LUSC | 1 | 0.79 | 1.2 | 0.42 |
| MESO | 0.74 | 0.22 | 0.96 | 0.91 |
| OV | 1 | 0.88 | 0.89 | 0.36 |
| PAAD | 1.1 | 0.78 | 1.4 | 0.15 |
| PCPG | 2 | 0.41 | 1.4 | 0.51 |
| PRAD | 0.78 | 0.7 | 0.91 | 0.67 |
| READ | 0.86 | 0.75 | 1.1 | 0.87 |
| SARC | 0.99 | 0.96 | 1.3 | 0.2 |
| SKCM | 1 | 0.81 | 1.1 | 0.38 |
| STAD | 1.1 | 0.71 | 1 | 0.97 |
| TGCT | 0.99 | 0.99 | 1.3 | 0.41 |
| THCA | 0.3 | 0.028 | 1 | 0.95 |
| THYM | 2.9 | 0.18 | 1 | 1 |
| UCEC | 0.97 | 0.94 | 1 | 0.94 |
| UCS | 2.2 | 0.025 | 1.9 | 0.078 |
| UVM | 1.1 | 0.84 | 1.4 | 0.49 |

Note: Group Cutoff; Median value
