# Supplementary material for: Comprehensive analysis on clinical significance and therapeutic targets of LDL receptor related protein 11 (LRP11) in liver hepatocellular carcinoma
Source: Front Pharmacol. 2024 Feb 15;15:1338929. doi: 10.3389/fphar.2024.1338929 (PMC10902445; doi:10.3389/fphar.2024.1338929)
Supplement: Supplementary file 2 [file Table3.DOCX]

**Supplementary Table S2.** Primer sequences for qRT-PCR.

| **Genes** | **Forward Primer (5’-3’)** | **Reverse Primer (5’-3’)** |
| --- | --- | --- |
| *hLRP11* | ACAGGAGGGAACCTACACCTTC | TGGTAGCGTGAGCAAGTGTGCA |
| *hTwist1* | GCCAGGTACATCGACTTCCTCT | TCCATCCTCCAGACCGAGAAGG |
| *hCDH2 (N-cadherin)* | CCTCCAGAGTTTACTGCCATGAC | GTAGGATCTCCGCCACTGATTC |
| *hZeb2* | AATGCACAGAGTGTGGCAAGGC | CTGCTGATGTGCGAACTGTAGG |
| *hFOXC1* | AGAAGGACAGGCTGCACCTCAA | GTTCTCGGTCTTGATGTCCTGG |
| *hFOXC2* | TCACCTTGAACGGCATCTACCAG | TGACGAAGCACTCGTTGAGCGA |
| *hVimentin* | AGGCAAAGCAGGAGTCCACTGA | ATCTGGCGTTCCAGGGACTCAT |
| *hTJP1 (ZO-1)* | GTCCAGAATCTCGGAAAAGTGCC | CTTTCAGCGCACCATACCAACC |
| *hDSP* | TGACAGACCGCTGGCAAAGGAT | GGCGTTTAGCATCATAGAGCCAC |
| *hGAPDH* | AACGGGAAGCTTGTCATCAATGGAAA | GCATCAGCAGAGGGGGCAGAG |
