# Supplementary material for: Comprehensive analysis on clinical significance and therapeutic targets of LDL receptor related protein 11 (LRP11) in liver hepatocellular carcinoma
Source: Front Pharmacol. 2024 Feb 15;15:1338929. doi: 10.3389/fphar.2024.1338929 (PMC10902445; doi:10.3389/fphar.2024.1338929)

**Supplementary Fig. 1.** Scatterplot of DNA methylation levels of LRP11 in samples of normal and HCC using SMART database. The probes located in CpG N_Shore (A), S_Shore (B), Island (C), and Open_Sea (D), respectively.

**A**


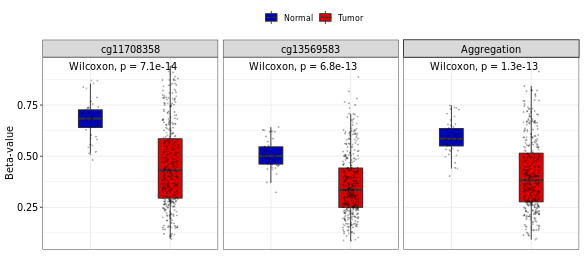


**B**


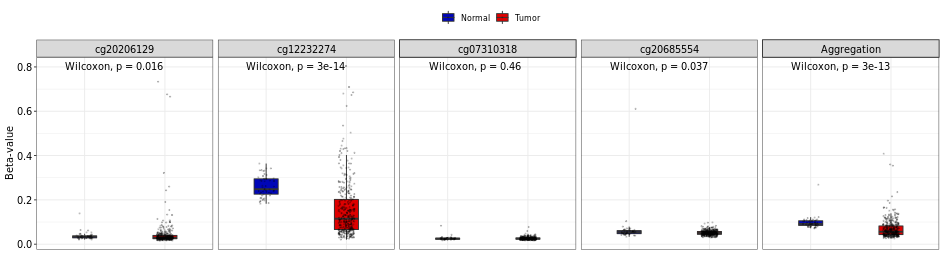


**C**


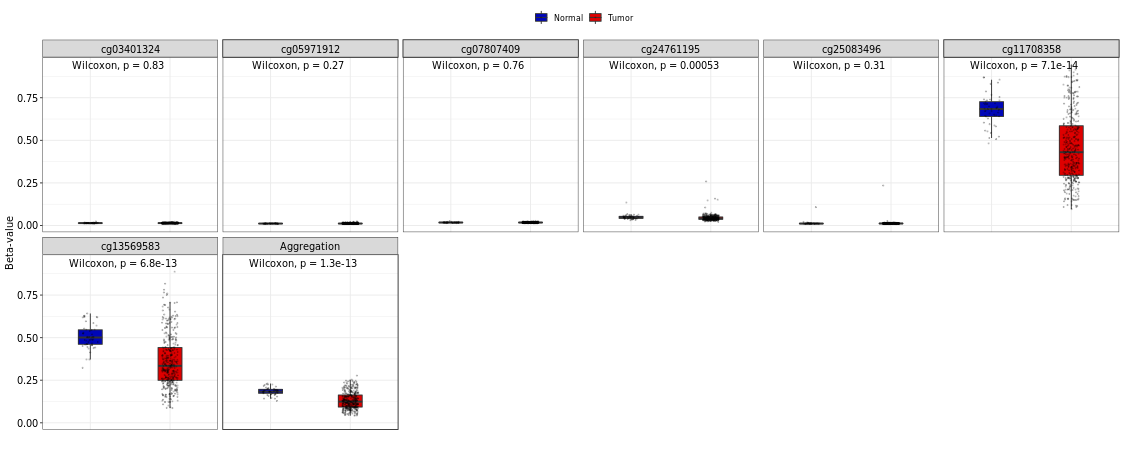


**D**


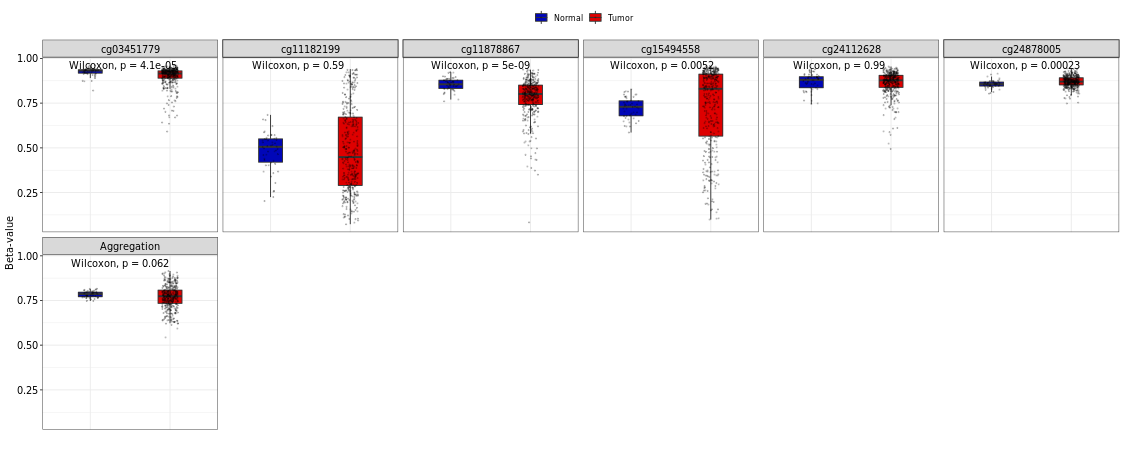


**Supplementary Fig. 2.** Spearman’s correlation between methylation level and mRNA levels of LRP11 in TCGA-LIHC using SMART database. The probes located in CpG N_Shore (A), S_Shore (B), Island (C), and Open_Sea (D), respectively.

**A**


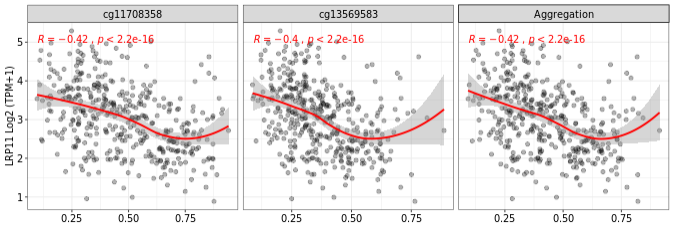


**B**


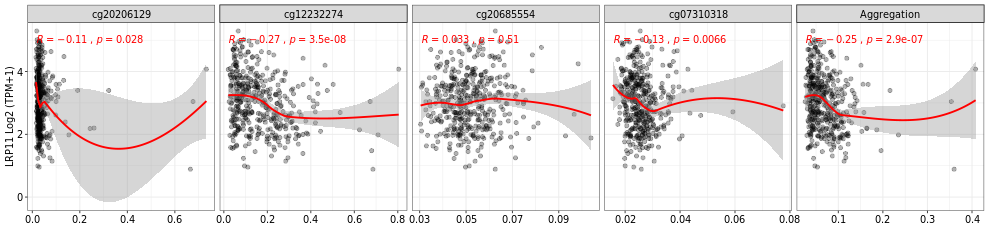


**C**


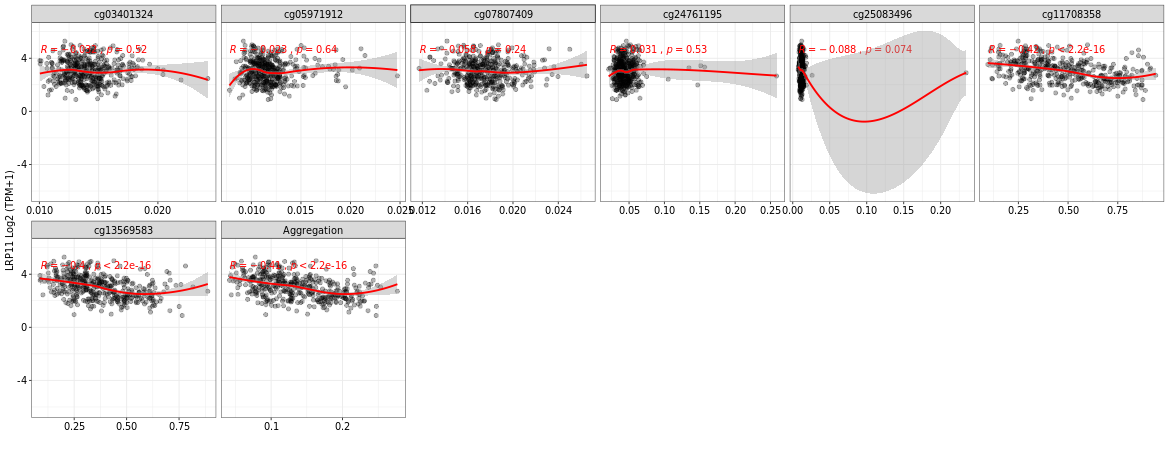


**D**


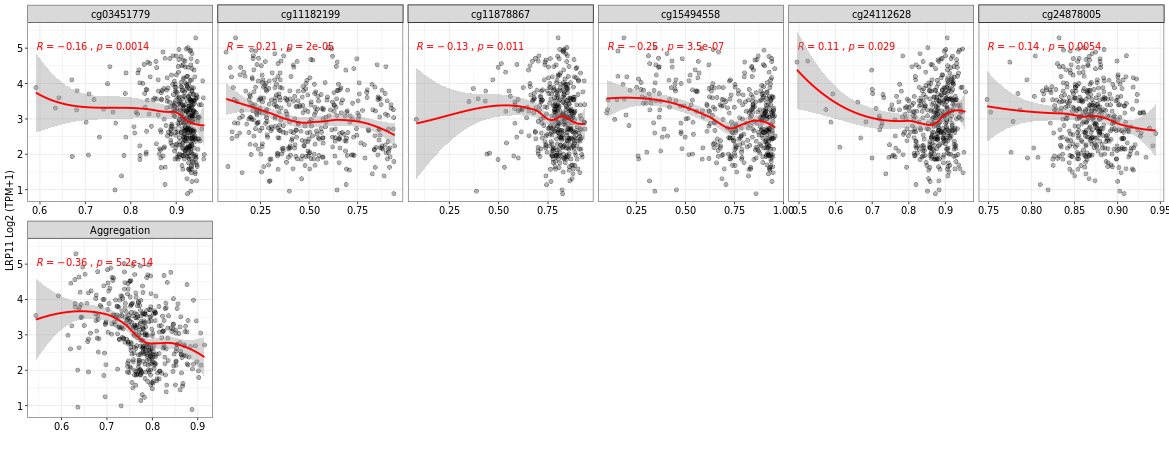

Supplement: Supplementary file 3 [file Table4.DOCX]
